# A Genome-Wide CRISPR Library for High-Throughput Genetic Screening in *Drosophila* Cells

**DOI:** 10.1016/j.jgg.2015.03.011

**Published:** 2015-06-20

**Authors:** Andrew R. Bassett, Lesheng Kong, Ji-Long Liu

**Affiliations:** a MRC Functional Genomics Unit, University of Oxford, Department of Physiology, Anatomy and Genetics, South Parks Road, Oxford, OX1 3PT, United Kingdom; b Genome Engineering Oxford, Sir William Dunn School of Pathology, University of Oxford, South Parks Road, Oxford, OX1 3RE, United Kingdom

**Keywords:** CRISPR/Cas9, Genome-wide library, *Drosophila*, Cas9, CRISPR associated protein 9, CRISPR, clustered regularly interspaced short palindromic repeat, HTS, high throughput sequencing, sgRNA, single guide RNA

## Abstract

The simplicity of the CRISPR/Cas9 system of genome engineering has opened up the possibility of performing genome-wide targeted mutagenesis in cell lines, enabling screening for cellular phenotypes resulting from genetic aberrations. *Drosophila* cells have proven to be highly effective in identifying genes involved in cellular processes through similar screens using partial knockdown by RNAi. This is in part due to the lower degree of redundancy between genes in this organism, whilst still maintaining highly conserved gene networks and orthologs of many human disease-causing genes. The ability of CRISPR to generate genetic loss of function mutations not only increases the magnitude of any effect over currently employed RNAi techniques, but allows analysis over longer periods of time which can be critical for certain phenotypes. In this study, we have designed and built a genome-wide CRISPR library covering 13,501 genes, among which 8989 genes are targeted by three or more independent single guide RNAs (sgRNAs). Moreover, we describe strategies to monitor the population of guide RNAs by high throughput sequencing (HTS). We hope that this library will provide an invaluable resource for the community to screen loss of function mutations for cellular phenotypes, and as a source of guide RNA designs for future studies.

## Introduction

RNA-guided endonucleases derived from the clustered regularly interspaced short palindromic repeat (CRISPR) adaptive immune system in bacteria have revolutionised our ability to generate site-specific mutations in many organisms (reviewed in Doudna and Charpentier, 2014), including *Drosophila* (Gratz et al., 2013; Bassett et al., 2013; Kondo and Ueda, 2013; Wei et al., 2013; Yu et al., 2013; Bassett and Liu, 2014; Gratz et al., 2014; Port et al., 2014; Ren et al., 2014; Sebo et al., 2014). The CRISPR associated (Cas9) endonuclease can be guided to its target site in the DNA by complimentary base pairing between the first 20 nt of the single guide RNA (sgRNA) and the genomic DNA (Brouns et al., 2008; Gasiunas et al., 2012; Jinek et al., 2012). The predictability and simplicity of this system coupled to oligonucleotide printing techniques makes it possible to design and produce large libraries of nucleases that target many tens of thousands of sites in a genome, for instance every protein-coding gene (Koike-Yusa et al., 2014; Shalem et al., 2014; Wang et al., 2014). These libraries of nucleases can be used for genetic screening for cellular phenotypes, and provide considerable improvements over currently employed RNA interference (RNAi) techniques in the specificity of target recognition (Ma et al., 2006; Koike-Yusa et al., 2014; Shalem et al., 2014; Wang et al., 2014). Importantly, CRISPR nucleases generate genetic mutations, which can remove gene function completely, and are stable during cell divisions, allowing analysis of phenotypes that only manifest after long periods of time.

*Drosophila* cell lines have been extensively employed for genome-wide RNAi screening for a variety of cellular phenotypes (reviewed in Boutros and Ahringer, 2008; Bakal, 2011). This has been in part due to the efficacy of RNAi in this cell line and the simplicity of its delivery. Furthermore, *Drosophila* also benefits from a lower degree of redundancy in gene function than mammalian systems, making the effect of manipulating a single gene more dramatic. In addition, the *Drosophila* genome maintains orthologues of around 75% of human disease causing genes (Reiter et al., 2001; Lloyd and Taylor, 2010), and numerous fundamental processes are highly conserved between the two species, making the results directly transferrable.

Here, we describe the design and production of a genome-wide CRISPR library for *Drosophila* cells, which contains 40,279 sgRNAs targeting 13,501 genes. We demonstrate methods for analysis of library screens using high throughput sequencing (HTS), and hope that this will be a valuable resource for the *Drosophila* community for cellular phenotype screening, and as a source of sgRNAs for functional studies *in vivo*.

## Results

### Library design

**Fig. 1:**
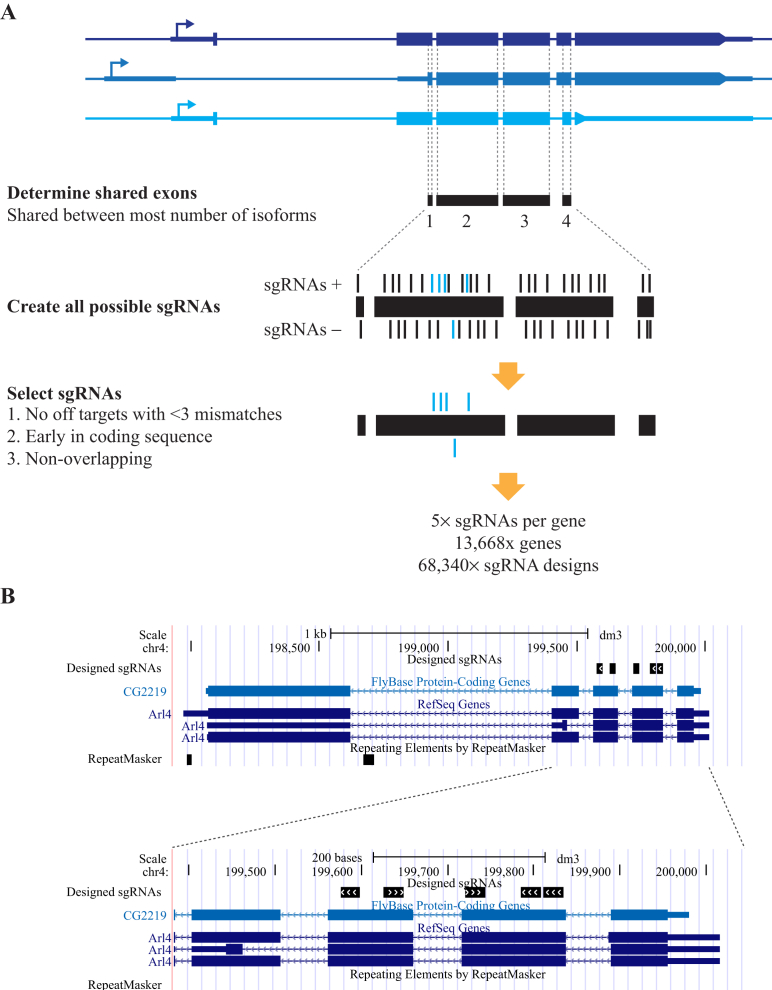
Design of a genome-wide sgRNA library. **A:** Strategy for library design. Fragments of coding exons shared between the maximum number of transcriptional isoforms were selected, and all possible sgRNAs of the format N_20_NGG were designed on both strands. Exons (blue boxes), transcriptional start sites (arrows) and untranslated regions (thick blue lines) are indicated. sgRNAs were selected based on the absence of any off-targets with less than three mismatches, and their position early in the coding sequence. Five non-overlapping sequences were selected. **B:** Example of designs. A screenshot from the UCSC browser shows designs for a typical gene.

The Cas9 protein induces a double strand break (DSB) at its complimentary target site in the genome that is repaired by the endogenous repair pathways in the cell (Rouet et al., 1994). In most cell types, non-homologous end joining (NHEJ) repair predominates over homologous recombination (HR) (Shrivastav et al., 2008). Since NHEJ is somewhat error-prone, this results in small insertion and deletion mutations at the cleavage site, which can be employed to cause frameshifts in protein-coding sequence (Bibikova et al., 2002). We therefore obtained the sequences of all protein coding exons from Flybase (release 5.57), and extracted exonic regions from those that are shared between the maximum number of transcripts in order to maximise the effect of the gene knockout (Fig. 1A).

In order for the Cas9 endonuclease to be effective, sgRNA target sites have to be followed by a protospacer adjacent motif (PAM) sequence (NGG) in the genome (Jinek et al., 2012). We computed all possible sgRNA target sites within the shared exonic regions on either DNA strand. These were ranked based on their position relative to the beginning of the coding sequence, since frameshifts early in the coding sequence are likely to have a more detrimental effect on protein function. Another consideration when designing sgRNAs is the potential for off-target mutagenesis (Hsu et al., 2013; Pattanayak et al., 2013; Doench et al., 2014; Frock et al., 2015; Tsai et al., 2015). To mitigate this problem, we mapped potential guide RNAs to all possible off-target sites in the *Drosophila* genome with up to 3 mismatches. Any sgRNAs that mapped to a potential off-target site were excluded, and five non-overlapping guide RNAs were selected for each gene. The cut off of 3 mismatches was chosen because of recent studies in *Drosophila*, which suggest that off-targets with more than 3 mismatches are highly unlikely to be mutagenized by the Cas9 protein (Gratz et al., 2014; Ren et al., 2014).

**Fig. 2:**
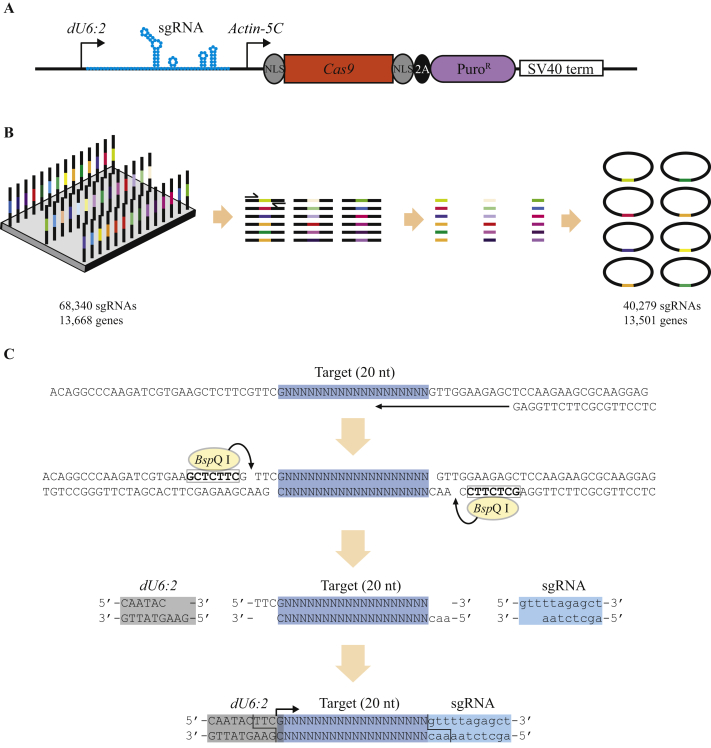
Cloning of sgRNA library. **A:** sgRNA expression vector. sgRNAs (blue) are expressed from a *Drosophila U6:2* promoter, along with the Cas9 protein from an *Actin-5C* promoter. Cas9 (red box) contains N- and C-terminal nuclear localisation sequences (NLS, grey oval), and is expressed as a bicistronic transcript with a puromycin N-acetyltransferase gene (purple oval) separated by a viral 2A peptide (black oval). An SV40 transcriptional terminator is also included (SV40 term). **B:** Oligo synthesis. sgRNA sequences were synthesised with common 5′ and 3′adaptors, and amplified by PCR followed by digestion with restriction enzymes and cloned into the expression vector. **C:** Cloning strategy for sgRNAs. The synthesised oligonucleotides were amplified by PCR using common adaptor sequences, and digested with the *Bsp*Q I restriction enzyme, followed by ligation into a similarly digested expression vector. The first base transcribed by the *dU6:2* promoter (G) is indicated by an arrow.

In total we designed 68,340 sgRNAs, covering 13,668 genes (approximately 78% of all *Drosophila* genes) and a typical distribution of these guides is indicated in Fig. 1B. These sgRNAs had common adaptors added, and were synthesised as a large pool, followed by PCR amplification using the common sequences, and digestion with a restriction enzyme to release the sgRNA sequences. These sequences were purified and cloned into an S2 expression vector (Bassett et al., 2014), which expresses the sgRNA from a *Drosophila U6:2* promoter, and the Cas9 protein with N- and C-terminal nuclear localisation signals under the control of the actin 5C promoter (Fig. 2A and B). Additionally, the vector contains a puromycin N-acetyltransferase gene to allow selection in S2 cells. Cloning of the sgRNAs uses the type IIS restriction enzyme *Bsp*Q I that allows scarless integration of the 20 nt target sequence (Fig. 2C). In order to maintain representation of the library, a total of approximately 7 million bacterial colonies were generated, representing at least a 100-fold coverage of the library.

### Library analysis

**Fig. 3:**
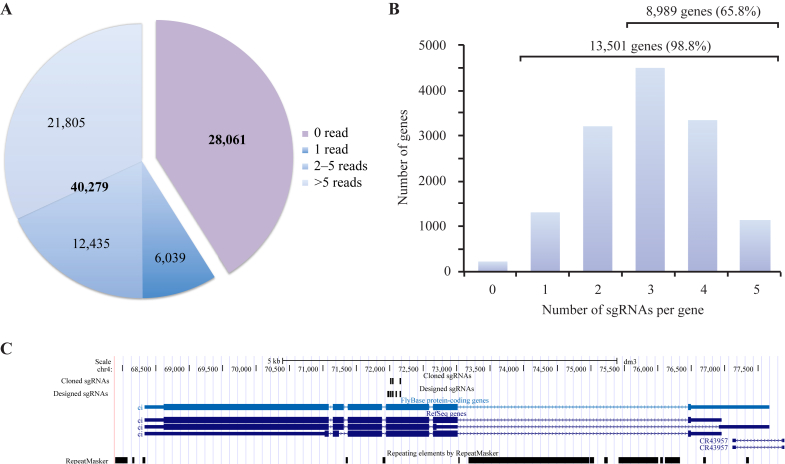
Analysis of cloned sgRNA library. **A:** Pie chart of the sgRNA sequences represented in the cloned library. Those sequences represented by 0 read (purple), 1 read (dark blue), 2–5 reads (mid blue) and more than 5 reads (light blue) are indicated. **B:** Histogram of the number of sgRNAs per gene in the cloned library. Total number of genes with designs is 13,668, and the number of genes with at least one sgRNA is 13,501. **C:** Example of cloned sgRNA. Screenshot from UCSC browser shows designed sgRNAs and cloned sgRNAs at a typical gene (CG2219).

We then assessed the coverage of sgRNAs in the library by high throughput sequencing (HTS) of a PCR product across the sgRNA sequences, at a depth of 827,527 mapped reads. This showed that 40,279 sgRNAs were represented by at least one sequencing read (Fig. 3A), and 21,805 sgRNAs by more than 5 reads. Analysis of the genes represented in the library showed that 13,501 genes (98.8%) were represented by at least one sgRNA and 8989 genes (65.8%) were targeted by 3 or more sgRNAs (Fig. 3B). This is of particular note since recent data suggest that the specificity of sgRNA screens can be improved by selecting those genes where similar effects are seen for multiple independent sgRNAs (Shalem et al., 2014; Koike-Yusa et al., 2014). A typical distribution of sgRNAs across a gene is shown in Fig. 3C, and BED files of both the total 68,340 sgRNA set and cloned 40,279 sgRNA set are available (Supplementary Data), which can be uploaded directly to a genome browser.

### Screening

**Fig. 4:**
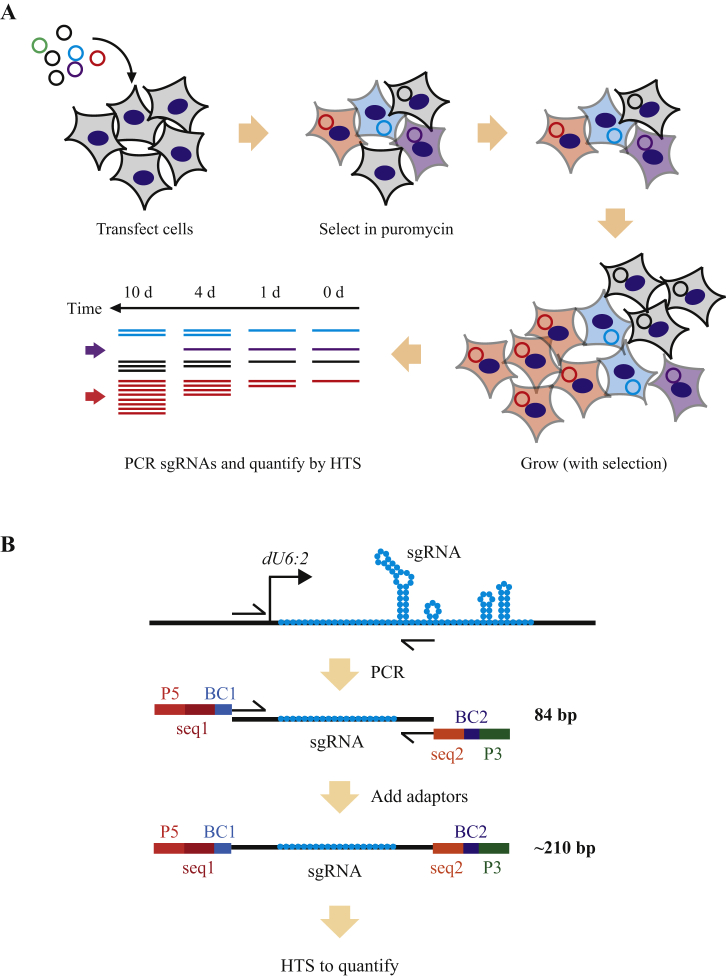
Screening and quantification strategy. **A:** Screening strategy. Cells were transfected with the library (coloured circles), and selected in puromycin to enrich for transfected cells followed by growth for 1, 4 and 10 days (d). sgRNAs were quantified by PCR and high throughput sequencing (HTS). **B:** Amplification of sgRNAs from cells. sgRNA sequences were amplified by PCR using common flanking sequences to obtain an 84 bp product. A second round of PCR was performed to add adaptors. These included the sequences required for amplification prior to sequencing (P5, light red, P3, green) and sequencing primer binding sites (seq1, dark red, seq2, orange) and included two barcodes (BC1, light blue, BC2, dark blue). BC1 is of variable length to increase sequencing library diversity.

In order to optimise protocols for screening using this library, we transfected S2R^+^ cells, selected in puromycin to enrich for transfected cells, and took samples of cells at various timepoints after selection (1, 4 and 10 days) (Fig. 4A). sgRNAs targeting genes which are essential for cell survival or growth would be expected to be depleted over time, and those important for cell death processes should show the opposite effect, and be enriched at later time points. We quantified the sgRNA population in each of these samples by PCR across the sgRNA sequences and HTS (Fig. 4B). A two-step strategy was used to first amplify the sgRNA sequence, and then add the appropriate adaptors for sequencing. In order to multiplex several libraries in one sequencing run, we added two barcodes, a variable length adaptor at the 5′ end to increase library complexity, and a second at the 3′ end.

Ideally, one sgRNA should be delivered per cell on average, so that we know that any effect within a cell is due to the sgRNA that it carries. If multiple sgRNAs were present in a cell, other guides could “piggy back” on the true hit, and dilute the signal. To optimise this variable, we diluted the library in carrier DNA that gave puromycin resistance but did not encode an sgRNA, at three different dilutions (undiluted (1:0), 10× dilution (1:10) and 100× dilution (1:100)).

**Fig. 5:**
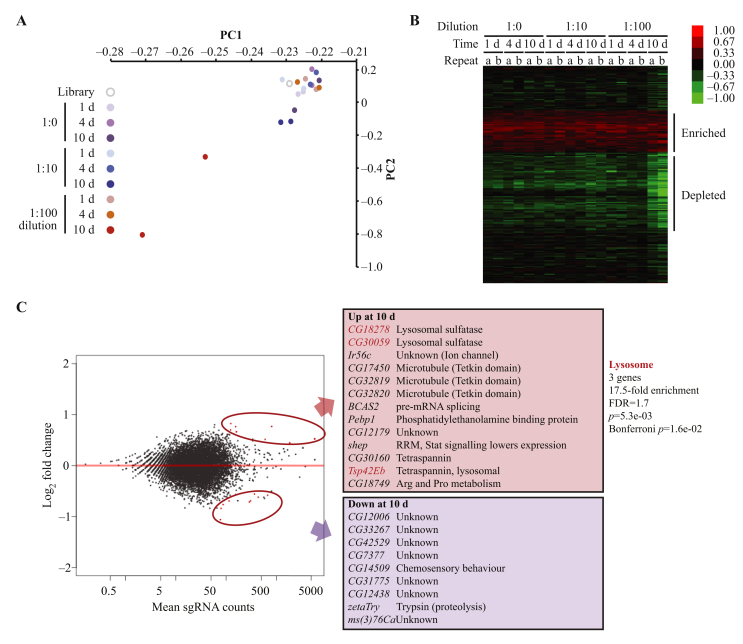
Optimisation of library screening conditions and pilot screen. **A:** Principal component analysis (PCA). sgRNA distributions in each condition were anlaysed by PCA. PC1 explained 98.6% and PC2 1.8% of the variance in the samples. **B:** Heat map of gene enrichment and depletion. Unsupervised hierarchical clustering of log_2_ fold change in sgRNA abundance for genes across different conditions. a and b correspond to biological replicates. Note that this does not include the entire gene set analysed, only the region that shows enrichment or depletion. **C:** Differential sgRNA abundance analysis. DESeq2 was used to identify statistically significant changes in sgRNA counts for all genes with ≥3 sgRNAs between samples at 1 day and 10 days (d) at a 1:100 dilution. MA plot (left panel) shows log_2_ fold change against sgRNA counts, with significant changes highlighted in red. Right panel shows the significantly enriched (red) or depleted (purple) genes, and functional enrichment as determined by DAVID. Lysosomal genes are indicated in bold red type.

We performed all transfections into around 20 million cells in biological duplicates, and analysed the sgRNA populations by principal component analysis (PCA) to look for clustering of the samples (Fig. 5A). This demonstrated that most of the samples clustered with the original, untransfected library. However, two samples were separated from the remainder, which represented the biological duplicates at the longest time point (10 days) and the highest library dilution (1:100). Consistent with this, if we analyse the samples at the longest time point (10 days) at the lower dilutions of library (1:10 and 1:1), they show a similar effect, but the magnitude is smaller. This suggests that the cells need to be grown for extended periods of time for the effects of gene disruption to be observed, and that higher library dilutions give a stronger signal to noise ratio.

In order to maximise any true signal, we analysed those 8989 genes that were targeted by 3 or more sgRNAs (Shalem et al., 2014; Koike-Yusa et al., 2014), since all sgRNAs for a gene should behave similarly. For each gene, the sgRNA counts were amalgamated into a single metric, and only those genes with 50 or more reads in the original library were analysed. We calculated the log_2_ fold change from the original untransfected library, and hierarchical clustering showed that there were two clusters of genes whose sgRNAs were enriched or depleted over time (Fig. 5B). This effect was most noticeable at later time points, as expected from the PCA. Analysis of functional enrichments in these two gene sets provided no significant enrichments. We analysed the differentially expressed genes between the early (1 day) and late (10 days) time points at a 1:100 dilution using DEseq2 (Fig. 5C), which showed a small number of statistically significant enrichments or depletions (red dots, gene lists, Fig. 5C). We analysed the functional annotations of these two gene sets to look for shared properties. Most of the genes whose sgRNAs were depleted over time (i.e., genes normally important for survival) did not have any functional annotations, but those genes whose sgRNAs were enriched over time were significantly enriched for those involved in lysosomal function. One explanation for this observation may be that mutations of these genes enhance cellular survival by preventing autophagy, but further experiments would be necessary to demonstrate this result. We also compared our results with a previous RNAi screen in *Drosophila* cells that analysed a similar viability phenotype, but found no significant overlap between the two analyses (Boutros et al., 2004).

## Discussion

Screening for cellular phenotypes is a powerful technique that has provided numerous insights into cell function and disease (reviewed in Boutros and Ahringer, 2008; Bakal, 2011). Targeted mutagenesis using the CRISPR/Cas9 system has several advantages over the current RNAi-based techniques in both its specificity (Ma et al., 2006; Shalem et al., 2014; Wang et al., 2014; Koike-Yusa et al., 2014), and the fact that it generates genetic mutations with a stronger effect, that is heritable and stable through cell division. This stability will enable more effective studies of synthetic lethality and other phenotypes such as epigenetic alterations that do not become apparent for many cell divisions and have therefore proven challenging with current RNAi technology.

We have designed and generated a genome-wide sgRNA library for *Drosophila*, and applied this to S2R^+^ cells to look for genes involved in cellular survival, cell competition or regulation of cell death. Although we identify a small number of significantly enriched or depleted sgRNAs, these are not enriched for known sets of essential genes, such as ribosomal genes, or apoptotic regulators, and do not overlap significantly with previously identified gene sets (Boutros et al., 2004). This may be due to that the cells have not been cultured for a sufficient time after inducing the mutations to observe any significant enrichment or depletion. This notion is consistent with the weak signal that we observe being present only at the longest (10 days) time point.

Another possibility may be that we are obtaining multiple sgRNAs in each cell, resulting in the dilution of the signal. In order to link the sgRNA present within a cell with its phenotype, each cell should ideally contain a single sgRNA. Our study suggests that as we increase dilution of the library in inert carrier DNA, we improve the signal obtained in the screen. It may also be possible that the signal:noise ratio would be further improved by greater dilutions, beyond the maximum that we use (1:100). In the future, it may also be beneficial to investigate alternative systems to ensure expression of only one sgRNA per cell, including viral-mediated transduction (Lee et al., 2000) or phiC31-mediated integration (Groth et al., 2004) of the sgRNA cassette.

We hope that our description of a genome-wide sgRNA library for *Drosophila* will enable the use of the CRISPR/Cas9 technique for screening in this powerful system, to investigate the molecular mechanisms of a variety of cellular functions. These may include fundamental processes such as cell cycle regulation, apoptosis, cell competition and DNA damage as well as identifying potential therapeutic targets for diseases such as cancer. Coupled with recent high-throughput assays to detect CRISPR-mediated cleavage (Frock et al., 2015; Tsai et al., 2015), our library provides a platform to perform large-scale testing of sgRNAs, which would provide an invaluable resource of tested sgRNAs that can be used for functional studies *in vivo*.

## Materials and methods

### Library design

Gene annotations were downloaded from Flybase (release 5.57), and CDS sequences were extracted. All manipulations were performed using custom Perl scripts unless otherwise stated. CDS sequences were used to extract exonic regions overlapping in as many transcript isoforms as possible. These were ordered by position relative to the beginning of the coding sequence, and off-targets were analysed by using Bowtie 1.1.1 (Langmead et al., 2009), to map to a custom index file containing all sequences in the *Drosophila* genome (dm3) of the form N20NGG or NAG with up to 3 mismatches. sgRNAs mapping multiple times were discarded, and 5 non-overlapping guides were selected for synthesis. Sequences of sgRNAs and mapping positions as a BED file are available in Supplementary Data.

### Library cloning

The 20 nt guide RNA target sites were appended with common adaptor sequences and had the first nucleotide substituted for a G, to improve transcription from the *U6:2* promoter. The final sequence was of the format shown in Fig. 2C. Oligonucleotide synthesis for the 68,340 sgRNA sequences was performed by CustomArray Inc, USA, and the assembled oligonucleotide pool was amplified using Phusion polymerase (NEB, UK) and oligonucleotides LibAmpF and LibAmpR (Table S1) with the following thermal cycling parameters (98°C 30 s, 30 cycles of (98°C 15 s, 55°C, 30 s, 72°C 30 s), 72°C 5 min). PCR products were purified using a PCR purification kit (Qiagen, UK), digested with *Bsp*Q I (NEB), and 20 nt fragments were extracted from a 20% acrylamide-TBE gel (Life Technologies, UK). Gel extraction was performed by homogenising gel pieces and overnight incubated in 600 μL 0.3 mol/L NaCl. DNA was purified by ethanol precipitation and cloned into pAc-sgRNA-Cas9 (Addgene, #49330, USA) also cut with *Bsp*Q I. Approximately 7 × 10^6^ transformants were obtained, corresponding to at least 100-fold coverage of the initial library. Colonies were scraped from bacterial plates and DNA was extracted using a maxiprep kit (Qiagen).

### Quantification of library

To quantify sgRNA abundance in the cloned library, sgRNA sequences were amplified by PCR using Phusion polymerase (NEB), oligonucleotides Screen_F2 and Screen_R (Table S1), and the following thermal cycling parameters (98°C 30 s, 30 cycles of (98°C 15 s, 60°C 30 s, 72°C 30 s), 72°C 5 min). PCR products were purified using a PCR purification kit (Qiagen), and a second round of PCR was performed to add on adaptors necessary for Illumina sequencing. For the cloned library, this was performed using ScreenampF0 and ScreenampR1 primers, Phusion polymerase (NEB) and the following thermal cycling parameters (98°C 30 s, 20 cycles of (98°C 15 s, 60°C 30 s, 72°C 30 s), 72°C 5 min). PCR products were gel extracted (Qiagen) from a 2% agarose gel to remove unincorporated primers and non-specific products followed by quantification and sequencing on an Illumina MiSeq (2 x 150 bp paired end reads).

### Library testing

Library transfections were performed into S2R^+^ cells (*Drosophila* Genomics Resource Centre #150) grown at 28°C in Schneider's medium (Sigma, UK) containing 10% foetal bovine serum (Life Technologies). Library plasmids were diluted 1:0, 1:10 or 1:100 in plasmid pAc-STABLE2-Puro (Addgene, USA, Dr James Sutherland) and transfected using Fugene HD (Roche, UK). 20 million cells were transfected in biological duplicates with 20 μg DNA and 60 μL Fugene. After 48 h, cells were collected by centrifugation at 500 g for 5 min, and replated in medium containing 5 μg/mL puromycin (Sigma). Samples of 20 million cells were taken at 1, 4 and 10 days after puromycin addition, and DNA was extracted with a Zymo Quick gDNA extraction kit (Zymo Research, UK). sgRNA sequences were amplified as above with oligonucleotides Screen_F2 and Screen_R using Taq polymerase (Bioline, UK) on the following thermal cycle (95°C 2 min, 30 cycles of (95°C 15 s, 60°C 30 s, 72°C 30 s), 72°C 5 min). The total volume of DNA extracted from 20 million cells was amplified using as many reactions as required (250 ng per 50 μL) to maintain complexity of the library. Reactions were pooled, and adaptors were added by a second round of PCR using 5 μL of the original PCR as template using Pfusion polymerase and the following thermal cycling parameters (98°C 30 s, 20 cycles of (98°C 15 s, 60°C 30 s, 72°C 30 s), 72°C 5 min). The samples were indexed as indicated in Table S2 using a variable length barcode on the forward primer (0–9 nt insertion) to increase library complexity, and a second barcode on the reverse primer (6 nt) to allow 18 samples to be sequenced together. PCR products were gel extracted (Qiagen) from a 2% agarose gel to remove unincorporated primers and non-specific products followed by quantification, pooling and sequencing on an Illumina MiSeq (2 × 150 bp paired end reads).

### Library analysis

FASTQ files were demultiplexed into individual libraries using a custom Perl script, and counts for each sgRNA were determined (raw data is available from the gene expression omnibus (GEO, accession number GSE67339) and processed data in Supplementary Data). Distributions of sgRNAs across genes were calculated using custom scripts. For PCA, counts were normalised according to total mapped library size, and analysed using R. To perform the gene-centered analyses, the 8989 genes with 3 or more sgRNAs were chosen, and the three or more sgRNA counts were summed across the gene (Supplementary Data). Hierarchical clustering was performed with Cluster 3.0 (de Hoon et al., 2004), and heat maps were generated using TreeView (Saldanha, 2004). Differential sgRNA expression was performed with DEseq2 (Love et al., 2014) using these counts on biological duplicates obtained from day 1 and day 10 at a 1:100 library dilution, and MA plots were generated using R. Functional enrichment analysis was performed with DAVID (Huang da et al., 2009), using the total 8989 gene set as background.

## Supplementary data

Table S1 Primers used for amplification and cloning of the library and for amplification, indexing and sequencing from cells.

Table S2 Metadata linking samples to indexes and sequencing primers.
