# Supplementary material for: A Genome-Wide CRISPR Library for High-Throughput Genetic Screening in Drosophila Cells
Source: J Genet Genomics. 2015 Jun 20;42(6):301–9. doi: 10.1016/j.jgg.2015.03.011 (PMC4508376; doi:10.1016/j.jgg.2015.03.011)
Supplement: Supplementary file 1 [file mmc1.docx]

| **Name** | **Sequence (5’-3’)** |
| --- | --- |
| **Library assembly** | |
| Library oligo | ACAGGCCCAAGATCGTGAA**GCTCTTC**GTTCGNNNNNNNNNNNNNNNNNNNGTTG**GAAGAGC**TCCAAGAAGCGCAAGGAG |
| LibAmpF | **gtgcagtgctacttatcgtg**ACAGGCCCAAGATCGTGAA**G** |
| LibAmpR | **cacgtagtgatcgctatgct**CTCCTTGCGCTTCTTGGA**G** |
| **Screen amplification and sequencing** | |
| Screen_F2 | TAGGTATGTTTTCCTCAATACTTCG |
| Screen_R | CGGACTAGCCTTATTTTAACTTGC |
| ScreenampF9 | AATGATACGGCGACCACCGAGATCT**ACACTCTTTCCCTACACGACGCTCTTCCGATCT***CGATGCCAC*TAGGTATGTTTTCCTCAATACTTCG |
| ScreenampF8 | AATGATACGGCGACCACCGAGATCT**ACACTCTTTCCCTACACGACGCTCTTCCGATCT***GATGCTAC*TAGGTATGTTTTCCTCAATACTTCG |
| ScreenampF7 | AATGATACGGCGACCACCGAGATCT**ACACTCTTTCCCTACACGACGCTCTTCCGATCT***ATGCTAG*TAGGTATGTTTTCCTCAATACTTCG |
| ScreenampF6 | AATGATACGGCGACCACCGAGATCT**ACACTCTTTCCCTACACGACGCTCTTCCGATCT***TCCAAG*TAGGTATGTTTTCCTCAATACTTCG |
| ScreenampF5 | AATGATACGGCGACCACCGAGATCT**ACACTCTTTCCCTACACGACGCTCTTCCGATCT***GCAGC*TAGGTATGTTTTCCTCAATACTTCG |
| ScreenampF4 | AATGATACGGCGACCACCGAGATCT**ACACTCTTTCCCTACACGACGCTCTTCCGATCT***CTGC*TAGGTATGTTTTCCTCAATACTTCG |
| ScreenampF3 | AATGATACGGCGACCACCGAGATCT**ACACTCTTTCCCTACACGACGCTCTTCCGATCT***TAC*TAGGTATGTTTTCCTCAATACTTCG |
| ScreenampF2 | AATGATACGGCGACCACCGAGATCT**ACACTCTTTCCCTACACGACGCTCTTCCGATCT***AG*TAGGTATGTTTTCCTCAATACTTCG |
| ScreenampF1 | AATGATACGGCGACCACCGAGATCT**ACACTCTTTCCCTACACGACGCTCTTCCGATCT***C*TAGGTATGTTTTCCTCAATACTTCG |
| ScreenampF0 | AATGATACGGCGACCACCGAGATCT**ACACTCTTTCCCTACACGACGCTCTTCCGATCT**TAGGTATGTTTTCCTCAATACTTCG |
| ScreenampR1 | CAAGCAGAAGACGGCATACGAGAT*ATCACG***GTGACTGGAGTTCAGACGTGTGCTCTTCCGATCT**CGGACTAGCCTTATTTTAACTTGC |
| ScreenampR2 | CAAGCAGAAGACGGCATACGAGAT*CGATGT***GTGACTGGAGTTCAGACGTGTGCTCTTCCGATCT**CGGACTAGCCTTATTTTAACTTGC |
