# Supplementary material for: A Genome-Wide CRISPR Library for High-Throughput Genetic Screening in Drosophila Cells
Source: J Genet Genomics. 2015 Jun 20;42(6):301–9. doi: 10.1016/j.jgg.2015.03.011 (PMC4508376; doi:10.1016/j.jgg.2015.03.011)
Supplement: Table S1 — Primers used for amplification and cloning of the library and for amplification, indexing and sequencing from cells. [file mmc2.docx]

| **Name** | **F primer** | **R primer** | **Description** |
| --- | --- | --- | --- |
| 0A | ScreenampF0 | ScreenampR1 | Raw library (pre-transfection) |
| 1A | ScreenampF1 | ScreenampR1 | 1:0 dilution, day 1, repeat 1 |
| 1B | ScreenampF1 | ScreenampR2 | 1:0 dilution, day 1, repeat 2 |
| 2A | ScreenampF2 | ScreenampR1 | 1:0 dilution, day 4, repeat 1 |
| 2B | ScreenampF2 | ScreenampR2 | 1:0 dilution, day 4, repeat 2 |
| 3A | ScreenampF3 | ScreenampR1 | 1:0 dilution, day 10, repeat 1 |
| 3B | ScreenampF3 | ScreenampR2 | 1:0 dilution, day 10, repeat 2 |
| 4A | ScreenampF4 | ScreenampR1 | 1:10 dilution, day 1, repeat 1 |
| 4B | ScreenampF4 | ScreenampR2 | 1:10 dilution, day 1, repeat 2 |
| 5A | ScreenampF5 | ScreenampR1 | 1:10 dilution, day 4, repeat 1 |
| 5B | ScreenampF5 | ScreenampR2 | 1:10 dilution, day 4, repeat 2 |
| 6A | ScreenampF6 | ScreenampR1 | 1:10 dilution, day 10, repeat 1 |
| 6B | ScreenampF6 | ScreenampR2 | 1:10 dilution, day 10, repeat 2 |
| 7A | ScreenampF7 | ScreenampR1 | 1:100 dilution, day 1, repeat 1 |
| 7B | ScreenampF7 | ScreenampR2 | 1:100 dilution, day 1, repeat 2 |
| 8A | ScreenampF8 | ScreenampR1 | 1:100 dilution, day 4, repeat 1 |
| 8B | ScreenampF8 | ScreenampR2 | 1:100 dilution, day 4, repeat 2 |
| 9A | ScreenampF9 | ScreenampR1 | 1:100 dilution, day 10, repeat 1 |
| 9B | ScreenampF9 | ScreenampR2 | 1:100 dilution, day 10, repeat 2 |
