# Supplementary material for: A Genome-Wide CRISPR Library for High-Throughput Genetic Screening in Drosophila Cells
Source: J Genet Genomics. 2015 Jun 20;42(6):301–9. doi: 10.1016/j.jgg.2015.03.011 (PMC4508376; doi:10.1016/j.jgg.2015.03.011)
Supplement: Table S2 — Metadata linking samples to indexes and sequencing primers. [file mmc3.docx]

**8. Supplementary data**

**Table S1**

Primers used for amplification and cloning of the library and for amplification, indexing and sequencing from cells.

**Table S2**

Metadata linking samples to indexes and sequencing primers

**Supplementary data files**

**1_sgRNA_library_designed.bed**

BED file describing sgRNA position and sequence. Can be uploaded to browsers such as UCSC.

**2_sgRNA_library_cloned.bed**

As sgRNA_library_designed.bed, but only including those sgRNAs that were cloned and present in the final library

**3_sgRNA_library_designed.out**

File describing exons targeted, genomic position and sequence of sgRNA

**4_sgRNA_library_designed.txt**

Raw text file of sgRNA library sent for oligonucleotide synthesis

**5_WTCHG_154382_1.fastq.gz (deposited to Gene Expression Omnibus (GEO), accession number GSE67339)**

FASTQ file containing all sequences from library quantification and analysis (also see Table S1). 150 nt forward sequencing read.

**6_sgRNA_counts.out**

Raw count table of all 68,340 sgRNAs in all sequencing libraries

**7_sgRNA_counts_normalised.out**

As above, but normalised to total matching reads

**8_sgRNAs.out**

Subset of sgRNA_counts.out table containing genes with 3 sgRNAs

**9_sgRNAs.out**

Subset of sgRNA_counts.out table containing genes with 4 sgRNAs

**10_sgRNAs.out**

Subset of sgRNA_counts.out table containing genes with 5 sgRNAs

**11_Gene_counts.out**

Raw count table containing the sum of all sgRNA reads targeting each gene containing 3 or more sgRNAs

**12_Gene_counts_normalised.out**

As above, but normalised to total matching reads
